# Isothermal and Nonisothermal Crystallization Kinetics of Poly(ε-caprolactone) Blended with a Novel Ionic Liquid, 1-Ethyl-3-propylimidazolium Bis(trifluoromethanesulfonyl)imide

**DOI:** 10.3390/polym10050543

**Published:** 2018-05-18

**Authors:** Chun-Ting Yang, Li-Ting Lee, Tzi-Yi Wu

**Affiliations:** 1 Department of Materials Science and Engineering, Feng Chia University, Taichung 40724, Taiwan; zipper0501@gmail.com; 2 Department of Chemical and Materials Engineering, National Yunlin University of Science and Technology, Yunlin 64002, Taiwan; wuty@gemail.yuntech.edu.tw

**Keywords:** crystallization kinetics, ionic liquids, biodegradable polymer, poly(ε-caprolactone), specific interactions

## Abstract

Recently, ionic liquids (ILs) and biodegradable polymers have become crucial functional materials in green sustainable science and technology. In this study, we investigated the influence of a novel IL, 1-ethyl-3-propylimidazolium bis(trifluoromethanesulfonyl)imide ([EPrI][TFSI]), on the crystallization kinetics of a widely studied biodegradable polymer, poly(ε-caprolactone) (PCL). To obtain a comprehensive understanding, both the isothermal and nonisothermal crystallization kinetics of the PCL blends were studied. Incorporating [EPrI][TFSI] reduced the isothermal and nonisothermal crystallization rates of PCL. Regarding isothermal crystallization, the small *k* and 1/*t*_0.5_ values of the blend, estimated using the Avrami equation, indicated that [EPrI][TFSI] decreased the rate of isothermal crystallization of PCL. The Mo model adequately described the nonisothermal crystallization kinetics of the blends. Increasing the [EPrI][TFSI] content caused the rate-related parameter *F*(*T*) to increase. This indicated that the crystallization rate of PCL decreased when [EPrI][TFSI] was incorporated. The spherulite appearance temperature of the blending sample was found to be lower than that of neat PCL under a constant cooling rate. The analysis of the effective activation energy proposed that the nonisothermal crystallization of PCL would not be favorited when the [EPrI][TFSI] was incorporated into the blends. The addition of [EPrI][TFSI] would not change the crystal structures of PCL according to the results of wide angle X-ray diffraction. Fourier transform infrared spectroscopy suggested that interactions occurred between [EPrI][TFSI] and PCL. The crystallization kinetics of PCL were inhibited when [EPrI][TFSI] was incorporated.

## 1. Introduction

With increasing concern regarding environmental protection, biodegradable polymers are attracting substantially more attention [1,2]. Biodegradable polymers can be classified into three types according to their mode of formation: natural, biosynthetic, and chemosynthetic [3,4]. Chemically synthesized poly(ε-caprolactone) (PCL) is among the most attractive and commonly used polymers due to its biocompatibility, biodegradability, favorable miscibility with other polymers, and low-temperature adhesiveness [5,6]. Furthermore, PCL can be degraded by the hydrolysis of its ester linkages in its polymer chain under physiological conditions [5]. PCL is an environmentally friendly food packaging material and is used in different biomedical applications, such as scaffolding, tissue engineering, and the controlled release of drugs [6,7]. However, its poor thermal stability, mechanical properties, and barrier properties to water and gases have restricted its application [8]. Therefore, techniques such as blending, copolymerization, and adding inorganic fillers are used to obtain PCL composites with satisfactory performance [9,10,11,12]. Relevant studies have been conducted on the blending systems and copolymers of PCL. Numerous researchers have synthesized PCL copolymers or blends, such as PCL/PEG [9], PCL/polysaccharides [10], and PCL/PVC [11], and have incorporated various functional groups into PCL [12].

The physical properties of a polymer are closely related to the polymer’s crystallization behavior [13,14]. In general, a polymer might be crystallized under isothermal and nonisothermal conditions in practical processing. Relevant kinetic characters are generally investigated to understand the mechanism and crystallization rate of polymeric materials. Isothermal crystallization kinetics are widely studied [15,16,17,18,19]. Insight regarding the crystallization of polymers can be obtained by theoretically analyzing the kinetic data. Nonisothermal crystallization of polymers is also an important area of study [20,21,22], and the behavior of polymeric samples is generally analyzed at a constant cooling rate. Studies and discussions focus on nonisothermal crystallization because this process closely represents the industrial processing conditions [23,24,25]. To control the rate of crystallization and obtain materials with superior physical properties, the kinetic studies should be performed with suitable models by using mathematical analysis [5].

Ionic liquids (ILs) are composed of organic cations and organic or inorganic anions [26,27]. At room temperature or temperatures approaching room temperature, ILs are usually liquid-like. ILs can be used as “green solvents” for dissolving polar and nonpolar organic compounds and inorganic chemicals. Generally, ILs possess properties such as nonflammability, negligible vapor pressure, high ionic conductivity, thermal stability, and a wide electrochemical window [28,29,30,31,32]. Among all types of ILs, the bis(trifluoromethanesulfonyl)imide-based ILs presents high thermal stability, a low melting point, and a wide liquid range. In addition, it has been found that intermolecular interactions occurred between the IL consisting of a imidazolium cation ring and a polymer with polar functional groups [33,34]. 1-Ethyl-3-propylimidazolium bis(trifluoromethanesulfonyl)imide ([EPrI][TFSI]) is a novel bis(trifluoromethanesulfonyl)imide-based IL containing an imidazolium cation [35]. [EPrI][TFSI] also presents a unique hydrophobic property and high thermal stability [35]. It can be expected that [EPrI][TFSI] can form intermolecular interactions with the polymer containing polar functional groups, and that their blends will be less sensitive to moisture.

To the best of our knowledge, blends of PCL with an IL have not yet been investigated. In this study, blends comprising a novel ionic liquid, [EPrI][TFSI], and PCL were analyzed. The aim of this study was to investigate the influence of an IL on the crystallization kinetics of PCL. The isothermal and nonisothermal kinetics of the [EPrI][TFSI]/PCL blends were thoroughly analyzed. We found that [EPrI][TFSI] influenced the crystallization kinetics of PCL in the blends. The isothermal and nonisothermal crystallization kinetics of PCL were inhibited when [EPrI][TFSI] was incorporated.

## 2. Materials and Methods

### 2.1. Materials and Blend Preparation

The PCL was obtained from Sigma-Aldrich (Sigma-Aldrich, St. Louis, MO, USA), and its molecular weight was 140,000 g/mol according to the manufacturer. [EPrI][TFSI] was synthesized from 1-ethyl-3-propylimidazolium bromide [35]. The detailed synthesis procedure is available in the literature [35]. [EPrI][TFSI]/PCL blends were prepared using the solution casting method. Tetrahydrofuran (THF) was used as the mutual solvent to prepare the blends. The film casting procedure was performed by evaporating the solvent at 45 °C, followed by vacuum drying at 60 °C for at least 48 h. Blend films with different compositions were obtained after evaporating the solvent completely.

### 2.2. Instruments and Experiments

We investigated the thermal behavior and crystallization of the [EPrI][TFSI]/PCL blends by using differential scanning calorimetry (DSC) (Perkin-Elmer DSC-8500, Perkin Elmer, Waltham, MA, USA). The thermal traces of the blends were obtained at a heating rate of 20 °C/min using a scan from −70 to 100 °C. The isothermal crystallization behavior of the blends was evaluated by first heating the samples to a temperature higher than the melting temperature of PCL (approximately 80 °C). The samples were then rapidly cooled to various crystallization temperatures (*T_c_*) for isothermal crystallization. Exothermal heat flow curves as a function of time were recorded to analyze the isothermal crystallization kinetics. The samples used for the study of nonisothermal crystallization were first heated to a temperature higher than the melting temperature of PCL, after which they were cooled at different cooling rates, and cooling traces were recorded.

The phase morphology and spherulite appearance were observed by a polarizing optical microscope (Olympus CX41, Olympus, Tokyo, Japan). The polarizing optical microscope used in this study was equipped with a Linkam THMS-600 microscopic hot stage. The samples for the optical microscopic observations were cast as thin films on glass slides and then dried thoroughly in a vacuum before characterization. To discuss the phase morphology of the blends, the specimens were heated on a hot stage, gradually increasing the temperature to the molten state. On the other hand, the spherulite appearance was observed by cooling the samples on hot stage at 1 °C/min from the melt.

The phase morphology of the [EPrI][TFSI]/PCL blends was also determined by using the scanning electron microscopy (SEM) (Hitachi S3000, Hitachi, Tokyo, Japan). A blend was solution-casted to produce a film that was sufficiently thick for convenient examination of the fracture surface of the cross-section. Prior to observation, the fractured blend films were coated with gold under the vacuum deposition.

The interactions in the blends were studied using Fourier transform infrared spectroscopy (FTIR) (Perkin-Elmer Frontier™, Perkin Elmer, Waltham, MA, USA). Every spectrum was collected at a resolution of 4 cm^−1^. The recorded spectra were all within the wavelength range of 400–4000 cm^−1^. The films used for FTIR measurements were prepared by coating a blend solution onto KBr pellets. The samples were then vacuum dried at 60 °C for at least 48 h to remove the residual solvent.

The crystalline structures of the blends were analyzed by the wide-angle X-ray diffraction (WAXD) (Bruker D2 PHASER, Bruker, Billerica, MA, USA) with copper kα radiation (30 kV and 10 mA). The samples were scanned under scanning 2θ angles between 5° and 50° with a step speed of 5 °C/min.

## 3. Results and Discussion

### 3.1. Phase Morphology and Thermal Analysis of the [EPrI][TFSI]/PCL Blends

**Figure 1:**
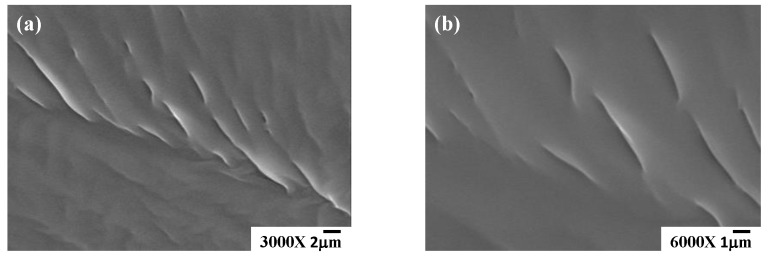
Images of scanning electron microscopy (SEM) for the 1-ethyl-3-propylimidazolium bis(trifluoromethanesulfonyl)imide/poly(ε-caprolactone) ([EPrI][TFSI]/PCL) blend at a composition of 20/80. The magnifications shown here are (**a**) 3000× and (**b**) 6000×.

In this research, the phase morphology and thermal behavior of [EPrI][TFSI]/PCL blends with different compositions were analyzed. A preliminary study for the phase morphology was made by the optical microscopy (OM) observation. The OM results are presented in Figure S1 in the Supplementary Materials. The OM results in Figure S1 demonstrate that the [EPrI][TFSI]/PCL blends have the same morphology type, as they present optically clear images in the amorphous molten state. The study of OM could preliminary suggest the phase homogeneity for the [EPrI][TFSI]/PCL blends. The phase morphology of the blends was further confirmed by using high-resolution scanning electron microscopy (HRSEM). The HRSEM image of the [EPrI][TFSI]/PCL = 20/80 blend was selected as the typical image for the demonstration. It should note that all blends revealed similar morphology types to the [EPrI][TFSI]/PCL = 20/80 blend. Figure 1 displays the SEM morphology of the [EPrI][TFSI]/PCL = 20/80 blend. A typical feature showing the fracture surface was resolved. In addition, as indicated in Figure 1, the high-resolution SEM image displayed the homogeneous morphology of the blend rather than the morphology with the phase separation. We further performed the SEM mapping analysis to detect the distribution of the constituent atoms in the area of SEM observation. We found that the result as a uniform distribution could be presented, suggesting a homogeneous mixing state for the constituent atoms in the blends. The result of mapping analysis is presented in Figure S2 in the Supplementary Materials. In short, with the results of SEM, it can confirm the phase homogeneity for the blends of [EPrI][TFSI] and PCL.

We also performed DSC to characterize the thermal behavior of the [EPrI][TFSI]/PCL blends. The samples used for DSC measurements were first melted at a temperature higher than the *T_m_* of PCL and then quenched for a sequential scan. The DSC heating scans for the blended samples after the melting/quenching treatment are displayed in Figure S3 of the Supplementary Materials. In Figure S3, the melting transitions of the blends are indicated. The glass transition temperatures of the blends could not be clearly determined by the DSC scans. This may be attributed to the resolution limit of the instrument used in the experiments. The important results of Figure S3 are summarized in Table 1. The melting enthalpy (Δ*H_m_*) and melting temperature (*T_m_*) of the [EPrI][TFSI]/PCL blends were estimated.

Table 1 provides the Δ*H_m_* and *T_m_* values of the [EPrI][TFSI]/PCL blends. Δ*H_m_* decreased as the [EPrI][TFSI] content increased in the blend. The Δ*H_m_* values decreased by 52.06–39.38 J/g with the addition of [EPrI][TFSI] to the blend. Moreover, *T_m_* also decreased with an increase in the [EPrI][TFSI] content. Therefore, the [EPrI][TFSI] content in the blends influenced the thermal properties of PCL. This may further suggest that the [EPrI][TFSI] was a molecular diluent in the blends and influenced the physical state of the polymer chain and the polymer’s physical properties such as its crystallization kinetics and melting point. Similar phenomena have been reported for other polymer–diluent pairs [36]. Generally, the presence of a diluent can reduce the equilibrium melting points (*T_m_*^0^) of a polymer. The value of *T_m_*^0^ represents the value of *T_m_* in the thermodynamically stable state. We confirmed the decrease in the *T_m_*^0^ of the [EPrI][TFSI]/PCL blends by estimating the *T_m_*^0^ of PCL in the blends using the classical Hoffman–Weeks method [37]. In this procedure, the measured *T_m_* of the specimens are plotted against the crystallization temperature *T_c_*, and the line *T_m_* = *T_c_* is extrapolated. The intercept of the line indicates the value of *T_m_*^0^. The specimens were crystallized at each *T_c_* for 8 h. Figure S4 in the Supplementary Materials displays the DSC heating traces of the blends at different values of *T_c_*, and Figure S5 in the Supplementary Materials illustrates Hoffman–Weeks plots of the [EPrI][TFSI]/PCL blends. The result of the Hoffman–Weeks plot is summarized in Table S1 of the Supplementary Materials. In the [EPrI][TFSI]/PCL blends, the *T_m_*^0^ of PCL decreased (from 70.34 to 66.49 °C) as the [EPrI][TFSI] content increased. Therefore, the [EPrI][TFSI] in the blends influenced the physical properties of PCL as a diluent. The isothermal and nonisothermal crystallization kinetics of the blends are discussed in the following sections to explore the influence of [EPrI][TFSI] on the crystallization kinetics and thermal behavior of PCL.

### 3.2. Isothermal Crystallization Kinetics of the [EPrI][TFSI]/PCL Blends

**Figure 2:**
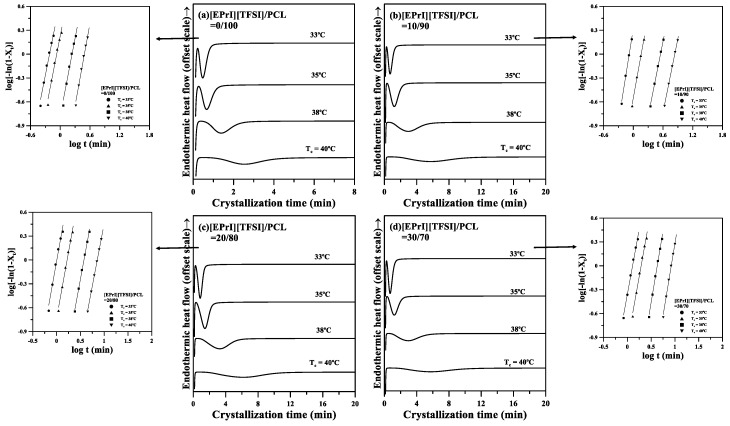
Differential scanning calorimetry (DSC) isothermal crystallization results of the [EPrI][TFSI]/PCL blends. The figures show the results for the blending compositions of (**a**) 0/100; (**b**) 10/90; (**c**) 20/80; and (**d**) 30/70. The inserts show the Avrami plots for the blends.

The isothermal crystallization thermograms for neat PCL and the [EPrI][TFSI]/PCL blends are presented in Figure 2. To perform isothermal crystallization, the blended samples were first heated to a temperature higher than the melting temperature of PCL and held at that temperature for 3 min to erase their thermal history. The samples were then rapidly cooled to various crystallization temperatures (*T_c_* = 33, 35, 38, and 40 °C).

**Figure 3:**
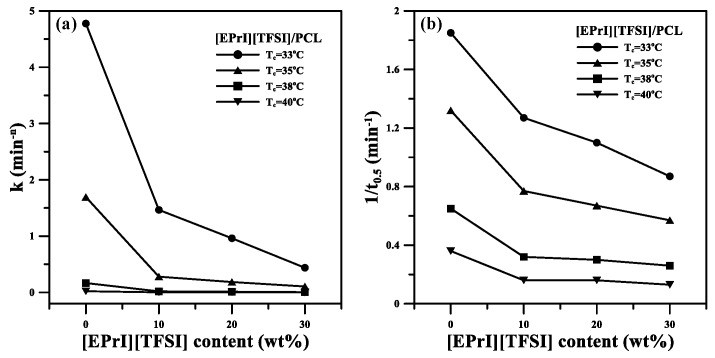
Plot of (**a**) *k* and (**b**) 1/*t*_0.5_ versus [EPrI][TFSI] content in the blends at different crystallization temperatures.

The Avrami equation [38,39] was applied to analyze the isothermal crystallization kinetics of the [EPrI][TFSI]/PCL blends. Theoretical background of the Avrami equation and its application to the analysis of the isothermal crystallization kinetics are summarized in the supplementary materials. The log–log representation of the Avrami equation was used to analyze the isothermal crystallization kinetics of the blends. The inserts of Figure 2 display the log–log representation of the Avrami plots for neat PCL and the [EPrI][TFSI]/PCL blends at different *T_c_*. The isothermal crystallization kinetics of neat PCL and the [EPrI][TFSI]/PCL blends are suitably described using the Avrami equation. From the inserts of Figure 2, the Avrami exponent (*n*) and crystallization rate constant (*k*) were calculated. The parameters estimated from the Avrami equation as well as the crystallization half-time (*t*_0.5_) and its reciprocal (1/*t*_0.5_) are listed in Table 2. The *n* values of neat PCL at the measured *T_c_* range are between 3 and 4, which are in agreement with the previous literature [40,41,42]. In addition, the average value of n for neat PCL and that of the blends are all close to 3.3. This result suggested that the addition of [EPrI][TFSI] did not considerably change the crystallization mechanism of PCL in the [EPrI][TFSI]/PCL blends. Based on the *n* values between 3 and 4, the crystallization mechanism for the blends may be considered as a three-dimensional truncated sphere growth [43]. It should also be noted that according to the Avrami theory, the *n* value for ideal three dimensional growth should be an integer without a fractional part. However, the *n* values of neat PCL and its blends do not satisfy the ideal state. The reason for this could be attributed to the slight difference between the actual process of growth and the ideal state. Since the samples for isothermal crystallization measurements were mostly confined to a thin film thickness in the DSC cells, the factor that the actual growth process may not be perfectly three-dimensional should be considered, and this factor might make a possible deviation from the ideal state. Similar results and statements have been also reported in recent literature [16]. In addition, it was also shown that *k* decreased as the [EPrI][TFSI] content increased. Furthermore, 1/*t*_0.5_ decreased as the [EPrI][TFSI] content increased. Generally, 1*/t*_0.5_ is a rate-dependent parameter. A larger 1/*t*_0.5_ indicates a higher crystallization rate. The values of *k* and 1/*t*_0.5_ decreased as the [EPrI][TFSI] content increased, which implied that the presence of [EPrI][TFSI] in the blends weakened the isothermal crystallization kinetics and decreased the crystallization rate of PCL. Figure 3a,b illustrated the variation in *k* and 1/*t*_0.5_, respectively, in the blends with different [EPrI][TFSI] content. Both *k* and 1/*t*_0.5_ gradually decreased as the [EPrI][TFSI] content increased. An increase in the [EPrI][TFSI] content of the blends reduced the isothermal crystallization rate of PCL.

### 3.3. Nonisothermal Crystallization Kinetics of the [EPrI][TFSI]/PCL Blends

**Figure 4:**
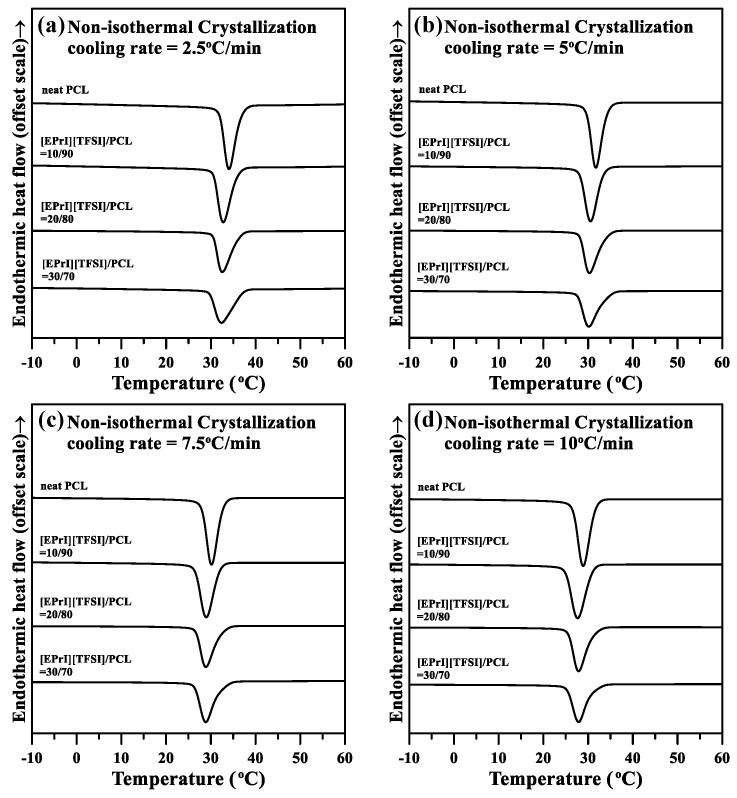
DSC results of nonisothermal crystallization at different cooling rates for different compositions of the [EPrI][TFSI]/PCL blends: (**a**) 0/100; (**b**) 10/90; (**c**) 20/80; and (**d**) 30/70.

**Figure 5:**
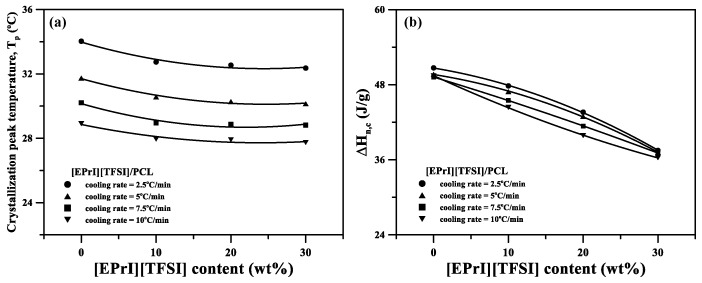
Plot of (**a**) *T_p_* and (**b**) *∆H_n,c_* versus [EPrI][TFSI] content at different cooling rates.

The nonisothermal crystallization kinetics of the [EPrI][TFSI]/PCL blends were studied to further understand the influence of [EPrI][TFSI] on the crystallization kinetics of PCL. Figure 4 displays the DSC results for neat PCL and the [EPrI][TFSI]/PCL blends at cooling rates of 2.5, 5, 7.5, and 10 °C/min. In Figure 5, we summarize the key information in Figure 4, which includes information related to the peak temperature (*T_p_*) and heat of the nonisothermal crystallization (Δ*H_n,c_*). Figure 5a displays the plot of *T_p_* versus [EPrI][TFSI] content at different cooling rates. According to Figure 5a, *T_p_* gradually shifted to a lower temperature as the [EPrI][TFSI] content increased, regardless of the cooling rate. Figure 5b presents plots of Δ*H_n,c_* versus [EPrI][TFSI] content at different cooling rates. Δ*H_n,c_* gradually decreased as the [EPrI][TFSI] content increased. The two aforementioned phenomena suggest that [EPrI][TFSI] retarded the nonisothermal crystallization of PCL in the blends.

**Figure 6:**
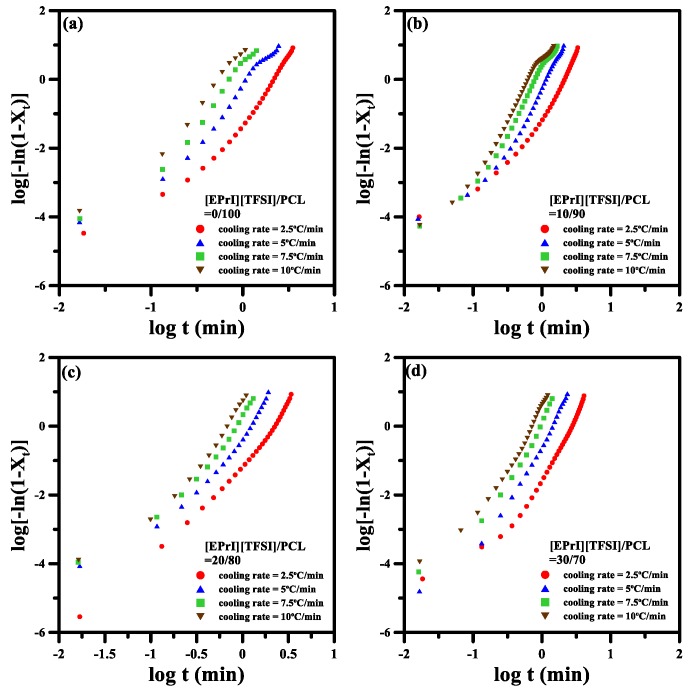
Avrami plots of log[−ln(1 − *X_t_*)] versus log(*t*) for the nonisothermal crystallization of the [EPrI][TFSI]/PCL blends. The figures show the results for the blending compositions of (**a**) 0/100; (**b**) 10/90; (**c**) 20/80; and (**d**) 30/70.

The nonisothermal crystallization kinetics of the [EPrI][TFSI]/PCL blends were further analyzed using three different models: the modified Avrami equation [44], Ozawa analysis [45], and Mo model [46]. Theoretical backgrounds of the abovementioned equations and their applications on the analysis of nonisothermal crystallization kinetics are summarized in the supplementary materials. Figure 6 presents logarithmic plots of the modified Avrami equation for the different [EPrI][TFSI]/PCL blends at various cooling rates. Each plot in Figure 6 exhibits a nonlinear relationship. Therefore, the modified Avrami equation was unsuitable for describing the nonisothermal crystallization kinetics of the [EPrI][TFSI]/PCL blends.

**Figure 7:**
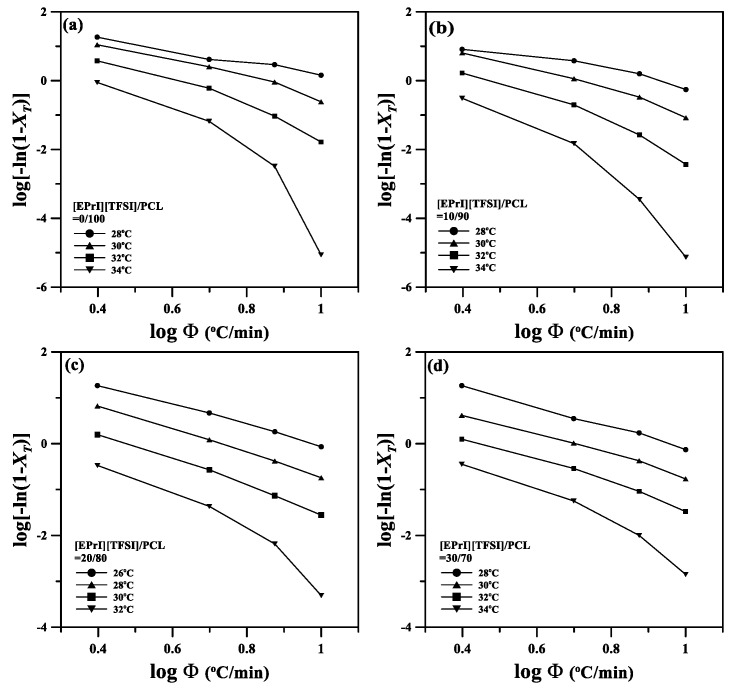
Ozawa plots of log[−ln(1 − *X_T_*)] versus log*Φ* for the nonisothermal crystallization of the [EPrI][TFSI]/PCL blends. The figures show the results for the blending compositions of (**a**) 0/100; (**b**) 10/90; (**c**) 20/80; and (**d**) 30/70.

The Ozawa equation was also applied to study the nonisothermal crystallization kinetics of the [EPrI][TFSI]/PCL blends. Figure 7 presents logarithmic plots of the Ozawa equation for the different [EPrI][TFSI]/PCL blends. All the plots in Figure 7 are also nonlinear. Thus, the Ozawa equation failed to describe the nonisothermal crystallization kinetics of the [EPrI][TFSI]/PCL blends.

As described in the aforementioned text, the modified Avrami equation and Ozawa equation could not adequately describe the nonisothermal crystallization kinetics of the [EPrI][TFSI]/PCL blends, as has also been indicated in previous studies [47,48]. This failure of the modified Avrami equation and Ozawa equation could be due to secondary crystallization in the blends [47,48].

**Figure 8:**
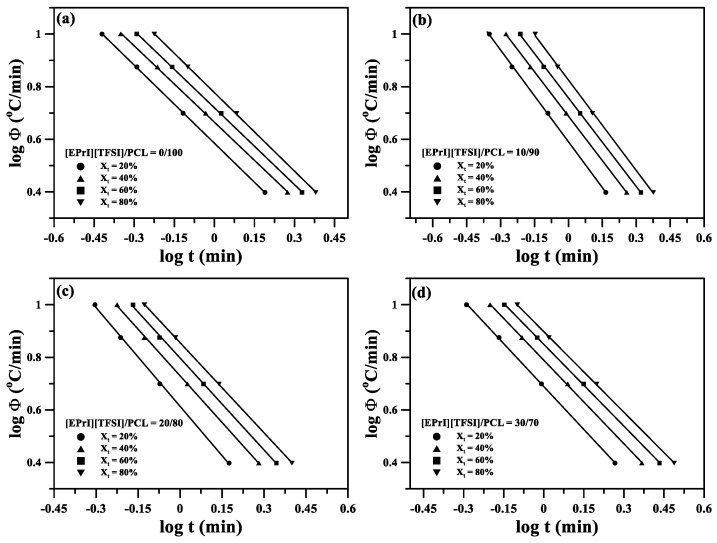
Mo model plots of log*Φ* versus log(*t*) for the nonisothermal crystallization of the [EPrI][TFSI]/PCL blends. The figures show the results for the blending compositions of (**a**) 0/100; (**b**) 10/90; (**c**) 20/80; and (**d**) 30/70.

In this study, we also applied the Mo model for studying the nonisothermal crystallization kinetics of the [EPrI][TFSI]/PCL blends. Figure 8 displays log*Φ* versus log*t* plots for the blends, and the plots are linear, which suggests that the Mo model is suitable for analyzing and describing the nonisothermal crystallization kinetics of the [EPrI][TFSI]/PCL blends. The nonisothermal crystallization parameters for the blends were estimated using the Mo model and are tabulated in Table 3. A high *F*(*T*) is associated with a low crystallization rate [49]. At the same degree of crystallinity, *F*(*T*) systematically increased with an increase in the [EPrI][TFSI] content of the blends, as indicated in Table 3. The Mo model analysis suggested that the presence of [EPrI][TFSI] in the blends decreased the nonisothermal crystallization rate of PCL. The isothermal and nonisothermal crystallization analyses thus indicated that [EPrI][TFSI] retarded both the isothermal and nonisothermal crystallization kinetics of PCL.

### 3.4. Polarizing Optical Microscopy (POM) Observations for [EPrI][TFSI]/PCL Blends under Cooling

**Figure 9:**
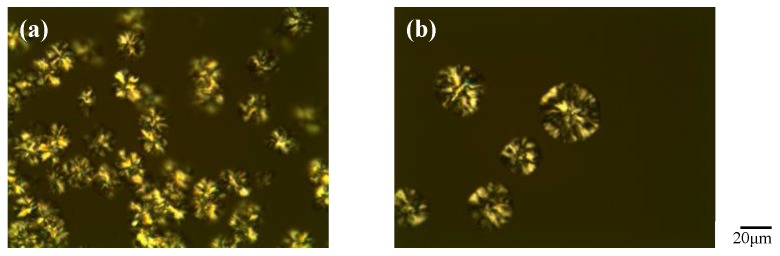
Polarizing optical microscopy (POM) pictures taken during the cooling process under the cooling rate of 1 °C/min for (**a**) neat PCL and (**b**) [EPrI][TFSI]/PCL = 20/80 blend.

Polarizing optical microscopy (POM) observations were also performed for the blends of [EPrI][TFSI] and PCL. By cooling at a constant rate from the molten state of PCL, we found that the spherulite appearance temperature of neat PCL and that of the [EPrI][TFSI]/PCL blends were different. The spherulite appearance temperatures of the blends were found to be lower than that of neat PCL. A demonstration of this can be found in Figure 9. As shown in Figure 9, for the [EPrI][TFSI]/PCL = 20/80 blend under a cooling rate of 1 °C/min, the growth of spherulites appeared at about 37 °C. On the other hand, for neat PCL, the growth of spherulites was found to start at a higher temperature, approximately 42 °C. This phenomenon is consistent with the *T_p_* results of the nonisothermal crystallization, as shown in the earlier section (Section 3.3). Both POM and DSC results of the nonisothermal crystallization demonstrated that the [EPrI][TFSI]/PCL blends crystallized at lower temperature compared to neat PCL, suggesting that the [EPrI][TFSI] retarded the nonisothermal crystallization of PCL in the blends.

### 3.5. Studies of Effective Ectivation Energy

**Figure 10:**
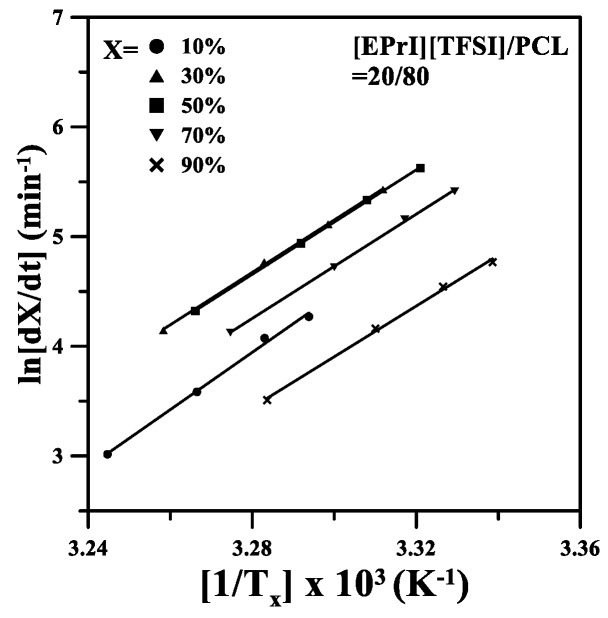
Friedman method plots of *ln*(*dX/dT*) versus 1/*T_X_* for the [EPrI][TFSI]/PCL = 20/80 blend.

**Figure 11:**
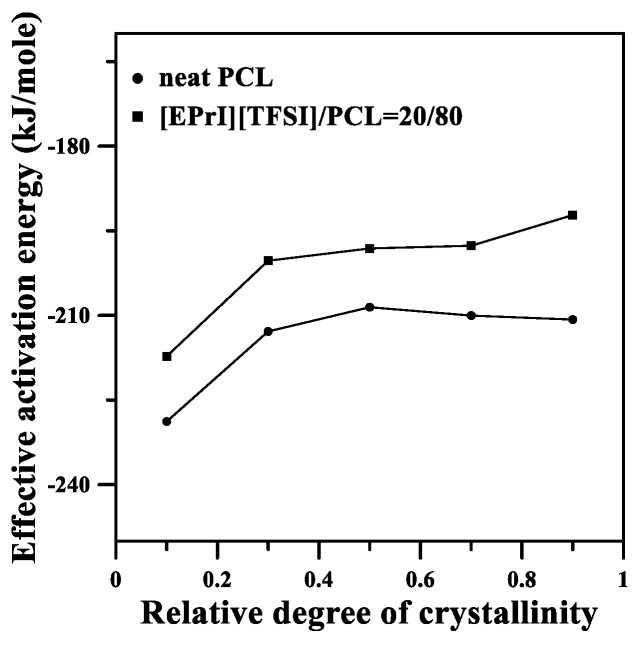
Effective activation energies for the neat PCL and the [EPrI][TFSI]/PCL = 20/80 blend estimated by the isoconversion Friedman method.

The effective activation energy was also discussed for the nonisothermal crystallization of the blends; it intended to study the influence on effective activation energy when [EPrI][TFSI] was incorporated into PCL. The isoconversion method of Friedman [50,51] was applied to our discussions. A detail description of the estimation of effective activation energy can be found in the supplementary materials. To demonstrate the important results of the Friedman estimation, the plots of *ln*(*dX*/*dT*) versus 1/*T_X_* for the [EPrI][TFSI]/PCL = 20/80 blend are shown in Figure 10 and the effective activation energy values of the neat PCL and [EPrI][TFSI]/PCL = 20/80 blend are demonstrated in Figure 11. In Figure 11, the plots show that the effective activation values of the [EPrI][TFSI]/PCL = 20/80 blend are smaller than that of the neat PCL, inferring that the nonisothermal crystallization of PCL would not be favorited with the addition of [EPrI][TFSI] in the blends.

### 3.6. Wide-Angle X-ray Diffraction (WAXD) Analyzes for Crystal Structures of [EPrI][TFSI]/PCL Blends

**Figure 12:**
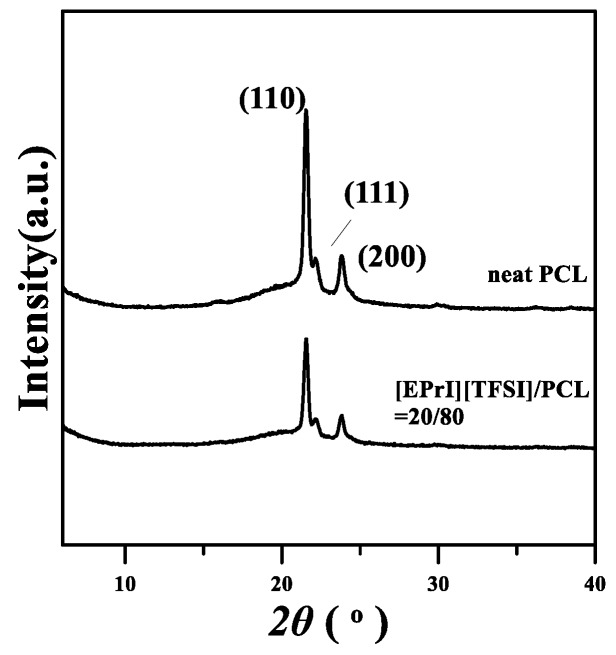
Wide-angle X-ray diffraction (WAXD) results of the neat PCL and the [EPrI][TFSI]/PCL = 20/80 blend. Samples subjected to the WAXD measurement were precrystallized at the *T_c_* = 38 °C.

Wide-angle X-ray diffraction (WAXD) technology was applied to discuss the crystal structures of the [EPrI][TFSI]/PCL blends. The samples for the WAXD study were isothermally crystallized at *T_c_* = 35 °C. Figure 12 demonstrates the results of WAXD for the neat PCL and the [EPrI][TFSI] = 20/80 blend. The results showed that neat PCL presented three main diffraction peaks at 2θ = 21.3°, 22°, and 23.8°, which are related to the reflection planes of (110), (111), and (200), respectively [52]. In addition, for the [EPrI][TFSI]/PCL = 20/80 blend, it showed the same reflection pattern as the neat PCL without any change in peak position or the appearance of new peaks. Relevant results revealed that the addition of [EPrI][TFSI] in PCL did not modify the crystal structures of PCL. In addition, the results obtained from the WAXD experiments also supported the abovementioned results of the isothermal crystallization kinetics (in Section 3.2), which demonstrated that the averaged Avrami *n* value of the neat PCL, and that of the [EPrI][TFSI]/PCL blends approached a similar value. The incorporation of [EPrI][TFSI] into PCL would not significantly influence the crystal growth mechanism of PCL, and therefore the crystal structures of PCL in the blends were not modified.

### 3.7. Possible Interactions between [EPrI][TFSI] and PCL in the Blends

**Figure 13:**
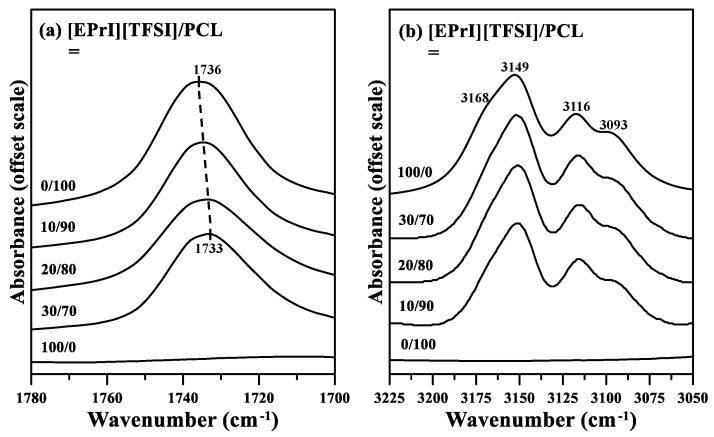
Fourier transform infrared (FTIR) spectra of the [EPrI][TFSI]/PCL blends indicating the (**a**) carbonyl stretching region (1780–1700 cm^−1^) and (**b**) C–H stretching vibration band (3225–3050 cm^−1^) of the imidazolium cation ring.

**Figure 14:**
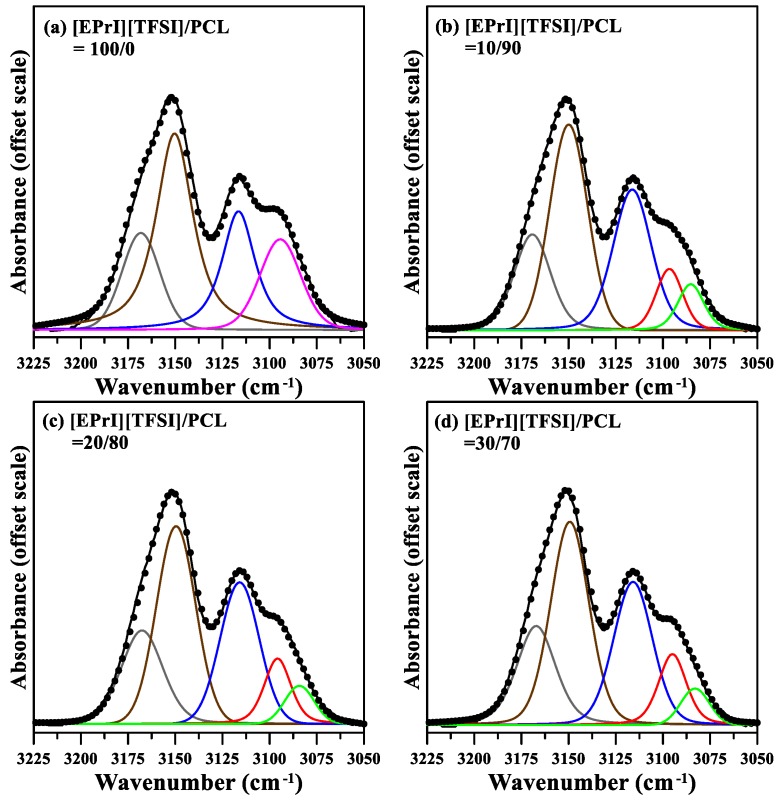
FTIR deconvolution results for the C–H stretching vibrational region of the imidazolium cation ring of the [EPrI][TFSI]/PCL blends. The figures show the results for the blending compositions of (**a**) 0/100; (**b**) 10/90; (**c**) 20/80; and (**d**) 30/70.

Our analyses of the crystallization kinetics of the blends indicated that [EPrI][TFSI] influenced the crystallization kinetics of PCL. Furthermore, we investigated the possible interactions between [EPrI][TFSI] and PCL in the blends. FTIR was used to provide spectral evidence of the interactions between [EPrI][TFSI] and PCL. The FTIR spectra of neat PCL, neat [EPrI][TFSI], and the [EPrI][TFSI]/PCL blends are displayed in Figure 13. Figure 13a displays the part of the IR spectra indicating the carbonyl stretching region (1780–1700 cm^−1^) of the [EPrI][TFSI]/PCL blends. The absorption peak of neat PCL occurred at 1736 cm^−1^. Furthermore, as the [EPrI][TFSI] content increased, the peak shifted to 1733 cm^−1^, which suggested the occurrence of an interaction between PCL and [EPrI][TFSI]. The samples were maintained in the molten amorphous state for measurements to avoid the complexity of crystallization. Figure 13b displays the part of the FTIR spectra indicating C–H stretching vibration (3225–3050 cm^−1^) in the imidazolium cation ring. The assignment of this absorption band has been previously described in the literature [53]. For neat [EPrI][TFSI], the spectrum contains a shoulder at 3168 cm^−1^ and three peaks at 3149 cm^−1^, 3116 cm^−1^, and 3093 cm^−1^. Spectral deconvolution was performed to obtain insight regarding the FTIR results for the imidazolium cation ring. Figure 14 presents the IR spectra for the deconvoluted C–H stretching vibration band of the imidazolium cation ring for the different [EPrI][TFSI]/PCL blends. The peak at 3093 cm^−1^ split into two peaks at 3087 cm^−1^ and 3097 cm^−1^. Similar results were observed for blends comprising ILs with an imidazolium ring cation [53]. As described in the literature [53], the splitting of the peak may have been due to (i) the complexation of the IL cations with polymer chains or (ii) the noncomplexation of the IL cations. The absorption bands at low (3087 cm^−1^) and high (3097 cm^−1^) wavenumbers can be attributed to the complexed and noncomplexed forms of the conformation, respectively. Therefore, a complex may have formed because of the interassociation between [EPrI][TFSI] and PCL. Nevertheless, the IR results suggested the occurrence of possible intermolecular interactions between [EPrI][TFSI] and PCL. The retarded crystallization kinetics of PCL in the [EPrI][TFSI]/PCL blends might be attributed to the interactions between [EPrI][TFSI] and PCL. The retardation of the crystallization kinetics caused by the intermolecular interactions has been also reported in the works discussing the blends containing crystalline polymer [54,55].

## 4. Conclusions

PCL is an attractive biodegradable polymer, and numerous studies have focused on its physical properties and blending system. In this study, blends comprising PCL and a novel IL, [EPrI][TFSI], were analyzed. We investigated the influence of [EPrI][TFSI] on the crystallization kinetics of PCL in the blends. The isothermal and nonisothermal crystallization kinetics of the [EPrI][TFSI]/PCL blends were evaluated. The isothermal crystallization kinetics were analyzed using the Avrami equation, and the results indicated that the presence of [EPrI][TFSI] reduced the isothermal crystallization rate of PCL. The *k* and 1/*t*_0.5_ values of neat PCL were smaller than those of the [EPrI][TFSI]/PCL blends. The nonisothermal crystallization of the [EPrI][TFSI]/PCL blends was suitably described using the Mo model. The *F*(*T*) values for the blends, estimated using the Mo model, were larger than those for neat the PCL. *F*(*T*) increased with an increase in the [EPrI][TFSI] content of the blend. This result indicated that the presence of [EPrI][TFSI] decreased the nonisothermal crystallization rate of PCL in the blends. The studies of POM showed that under a constant cooling rate, the spherulite appearance temperature of the blending sample was higher than that of the neat PCL. The analysis of the effective activation energy supposed that the nonisothermal crystallization of PCL would not be favorited when the [EPrI][TFSI] was incorporated into the blends. The WAXD discussions indicated that the addition of [EPrI][TFSI] would not change the crystal structures of PCL. In this study, we determined that the addition of [EPrI][TFSI] to PCL weakened both the isothermal and nonisothermal crystallization kinetics of PCL. The FTIR results suggested the formation of intermolecular interactions between [EPrI][TFSI] and PCL in the blends. The retardation of the crystallization kinetics of PCL in the blends could be attributed to the interactions between PCL and [EPrI][TFSI]. The influence of [EPrI][TFSI] on the crystallization kinetics of biodegradable PCL is thoroughly reported in this paper.

## Figures and Tables

**Table 1:** Δ*H_m_* and *T_m_* values of the [EPrI][TFSI]/PCL blends.

[EPrI][TFSI]/PCL (wt %)	Δ*H_m_* (J/g)	*T_m_* (°C)
0/100	52.06	56.83
10/90	49.61	55.76
20/80	43.04	54.83
30/70	39.38	53.18

**Table 2:** Parameters estimated from the isothermal crystallization results of the Avrami equation.

[EPrI][TFSI]/PCL (wt %)	*T_c_* (°C)	*n*	*k* (min^−*n*^)	*t*_0.5_ (min)	1/*t*_0.5_ (min^−1^)
0/100	33	3.14	4.775	0.54	1.85
	35	3.21	1.694	0.76	1.32
	38	3.26	0.167	1.55	0.65
	40	3.44	0.021	2.76	0.36
10/90	33	3.10	1.466	0.79	1.27
	35	3.45	0.28	1.29	0.77
	38	3.12	0.019	3.15	0.32
	40	3.08	2.6 × 10^−3^	6.09	0.16
20/80	33	3.35	0.962	0.91	1.10
	35	3.32	0.185	1.49	0.67
	38	3.26	0.013	3.39	0.30
	40	3.15	2.046 × 10^−3^	6.36	0.16
30/70	33	3.28	0.438	1.15	0.87
	35	3.36	0.106	1.75	0.57
	38	3.58	5.585 × 10^−3^	3.85	0.26
	40	3.63	4.406 × 10^−4^	7.60	0.13

**Table 3:** Nonisothermal crystallization parameters of the [EPrI][TFSI]/PCL blends, estimated using the Mo model.

[EPrI][TFSI]/PCL (wt %)	*X_t_* (%)	*a*	*F*(*T*)
0/100	20	0.99	3.80
	40	0.97	4.62
	60	0.97	5.25
	80	1.00	5.99
10/90	20	1.16	3.90
	40	1.13	4.87
	60	1.12	5.72
	80	1.15	6.69
20/80	20	1.25	4.11
	40	1.18	5.37
	60	1.17	6.27
	80	1.14	7.18
30/70	20	1.09	4.88
	40	1.06	6.15
	60	1.04	7.07
	80	1.02	7.90

## Supplementary Materials

The following are available online at http://www.mdpi.com/2073-4360/10/5/543/s1, Theoretical Backgrounds and Methods, Figure S1: Optical microscopy (OM) images of the [EPrI][TFSI]/PCL blends; Figure S2: Scanning electron microscopy (SEM) mapping results for the [EPrI][TFSI]/PCL = 20/80 blend; Figure S3: Differential scanning calorimetry (DSC) heating scans for the [EPrI][TFSI]/PCL blends after the melting/quenching treatment. Blends showing various compositions (wt %) were detected; Figure S4: DSC heating traces at different *T_c_* of the [EPrI][TFSI]/PCL blends with compositions (a) 0/100, (b) 10/90, (c) 20/80, and (d) 30/70; Figure S5: Hoffman–Weeks plots of the [EPrI][TFSI]/PCL blends; Table S1: Equilibrium melting points of the [EPrI][TFSI]/PCL blends with different compositions; References of Supplementary Materials.
