# Supplementary material for: Isothermal and Nonisothermal Crystallization Kinetics of Poly(ε-caprolactone) Blended with a Novel Ionic Liquid, 1-Ethyl-3-propylimidazolium Bis(trifluoromethanesulfonyl)imide
Source: Polymers (Basel). 2018 May 18;10(5):543. doi: 10.3390/polym10050543 (PMC6415448; doi:10.3390/polym10050543)
Supplement: Supplementary file 1 [file polymers-10-00543-s001.pdf]

Supplementary Materials

Isothermal and Nonisothermal Crystallization Kinetics of Poly(ε-caprolactone) Blended with a Novel Ionic Liquid, 1-Ethyl-3-propylimidazolium Bis(trifluoromethanesulfonyl)imide

Chun-Ting Yang [1], Li-Ting Lee [1,*] and Tzi-Yi Wu [2]

[1] Department of Materials Science and Engineering, Feng Chia University, Taichung 40724, Taiwan; zipper0501@gmail.com

[2] Department of Chemical and Materials Engineering, National Yunlin University of Science and Technology, Yunlin 64002, Taiwan; wuty@gemail.yuntech.edu.tw

* Correspondence: ltlee@fcu.edu.tw; Tel.: +886-4-2451-7250 (ext. 5306)

1. Theoretical Backgrounds and Methods

Isothermal Crystallization Kinetics

The Avrami equation [1,2] was used to evaluate the isothermal crystallization kinetics of the [EPrI][TFSI]/PCL blends. The following equation presents the original form of the Avrami equation.

$$ln(1 - X_t) = -kt^n \quad (1)$$

In equation (1), it should note the relative degree of crystallinity at time t is denoted by X_t. The value n is a constant relating to the crystallization mechanism. In addition, the rate constant is denoted by k in equation (1). The Avrami equation can be further converted into the log–log representation, and this representation can be applied to evaluate the relevant kinetic parameters. Equation (2) shows the Avrami equation in the log–log form.

$$log[- ln(1 - X_t)] = logk + nlogt \quad (2)$$

According to equation (2), it can estimate the values of n and k from the slope and intercept, respectively, of the log[−ln(1−X_t)] versus log(t) plot with a linear relationship. Moreover, it can also determine the crystallization half-time ($t_{0.5}$) according to the form shown in equation (3). It should note that the $t_{0.5}$ is the time at which the extent of crystallization is 50%

$$t_{0.5} = \left(\frac{ln2}{k}\right)^{1/n} \quad (3)$$

Non-isothermal Crystallization Kinetics

The non-isothermal crystallization kinetics of the blends comprising [EPrI][TFSI] and PCL was also discussed in this study. The influence of [EPrI][TFSI] on the non-isothermal crystallization of PCL can be further identified with relevant discussions. We applied several conventional models such as the modified Avrami equation [3], Ozawa equation [4], and Mo model [5] for our analysis. Equation (4) shows the modified Avrami equation [3] for the non-isothermal crystallization, and it can be correlated to the overall rate of non-isothermal crystallization.

$$X_t = 1 - exp\left(-Z_t t^{n'}\right) \quad (4)$$

In the modified Avrami equation [3] for the non-isothermal crystallization, it assumes that a series of infinitesimally small isothermal crystallization steps are involved in the crystallization process when the cooling rate is fixed. The exponent n' is related to the nucleation type and the dimension of the crystal growth. Furthermore, Z_t is a rate constant and can be correlated to the parameters of nucleation and growth. The time (t) and temperature (T) relationship of the non-isothermal crystallization can be further derived as the following equation.

$$t = \frac{T_0 - T}{\Phi} \quad (5)$$

In equation (5), the rate of cooling is denoted by Φ. The modified Avrami equation in the logarithmic form for analyzing non-isothermal crystallization kinetics is presented as the equation (6).

$$log[-ln(1 - X_t)] = logZ_t + nlogt \quad (6)$$

Ozawa [4] further modified the Avrami equation. Ozawa suggested that numerous infinitesimal steps of isothermal crystallization are included in the crystallization process proceeding non-isothermally. The Ozawa equation for analyzing non-isothermal crystallization is shown as the equation (7).

$$X_T = 1 - exp\left(-\frac{K(T)}{\Phi^m}\right) \quad (7)$$

In equation (7), the relative crystallinity (at temperature T) and the cooling function related to the overall rate of crystallization are denoted by X_T and $K(T)$, respectively. The cooling rate and the Ozawa exponent are denoted by Φ and m, respectively, in equation (7). The crystal growth dimension is associated with the Ozawa exponent. By taking the logarithm of both sides of equation (7), the Ozawa equation can be rearranged, and the logarithmic form of the Ozawa equation can be presented. The Ozawa equation in the logarithmic form is shown as equation (8).

$$log[-ln(1 - X_T)] = logK(T) - mlog\Phi \quad (8)$$

The Mo model derived by Mo and his coworkers [5] were also applied for analyzing the non-isothermal crystallization in this study. The Mo equation is shown as the following equation.

$$log\Phi = -alogt + logF(T) \quad (9)$$

In equation (9), the correlation between the cooling rate and crystallization time is demonstrated. The Mo index is denoted by a in equation (9), and this index is equal to the ratio of Avrami exponent (n)/Ozawa exponent (m). In addition, the log$F(T)$ in equation (9) is equal to the value of $[K(T)/k]^{1/m}$. At a certain temperature in unit crystallization time, it can associate the value of $F(T)$ with the cooling rate needed to reach a defined degree of crystallinity. The parameter $F(T)$ can be estimated from the intercept of the logΦ versus log(t) plot at a defined relative crystallinity. In addition in the same plot of logΦ versus log(t), it can determine the value of a by the slop of logΦ versus log(t) plot.

Effective Activation Energy of Non-isothermal Crystallization

The effective activation energy were also estimated for the neat PCL and the [EPrI][TFSI]/PCL blend. We used the isoconversion method proposed by Friedman [6,7] for relevant estimations. According to the Friedman method, the equation relating to the effective activation energy can be shown as equation 10,

$$ln(\frac{dX}{dt})_{X,i} = Constant - \frac{\Delta E_X}{RT_{X,i}} \quad (10)$$

In equation 10, at a specific conversion X, dX/dt represents the instantaneous crystallization rate as a function of time. ΔE_X is the effective activation energy at a specific conversion X and $T_{X,i}$ represents the temperatures correlated with a specific X at different cooling rates. The subscript i means each cooling rate used in the non-isothermal crystallization experiment. To estimate the effective activation energy, the X as a function of t can be obtained from the non-isothermal crystallization results. The value of dX/dt at a given X can be calculated by differentiating the obtained $X(t)$ function. The specific T_X value related to a given X can be further found according to the relationship among X, t, and T. By plotting the specific value of ln(dX/dt) to $1/T_X$, the slope with a straight line plot can be used to estimate the effective activation energy ΔE_X. More detail descriptions on the estimation of the effective activation energy by the Friedman method can be found in the literatures [7].

2. Results and Figures

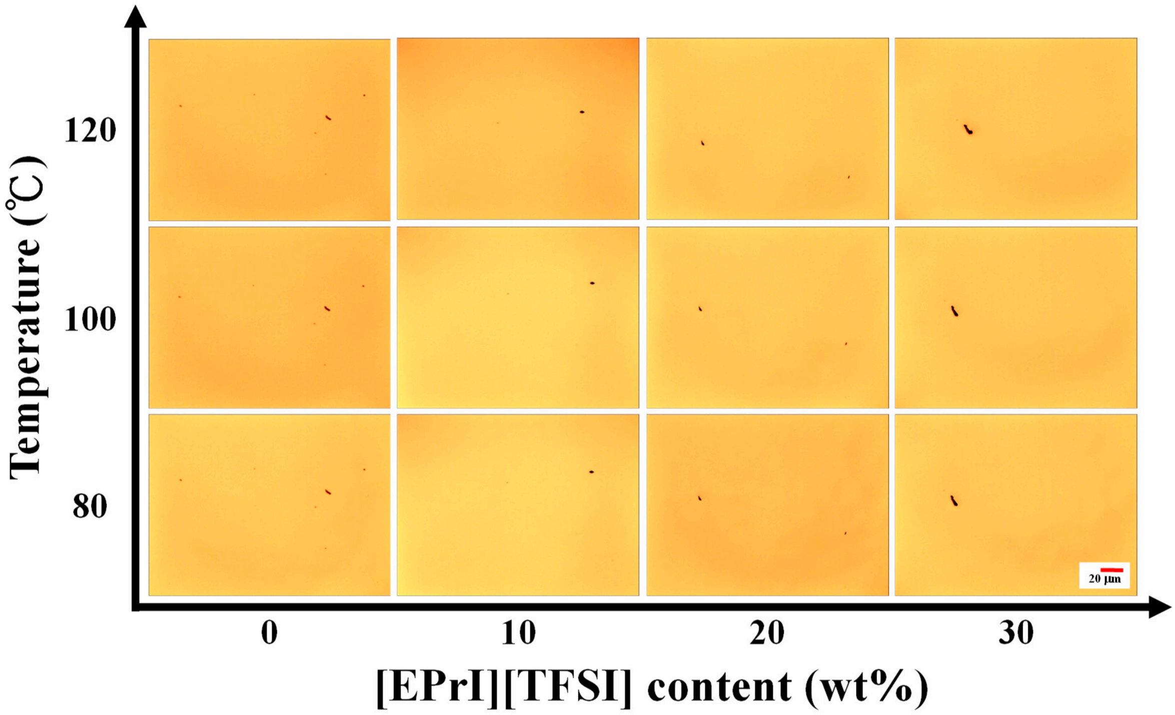

Figure S1. Optical microscopy (OM) images of the [EPrI][TFSI]/PCL blends.

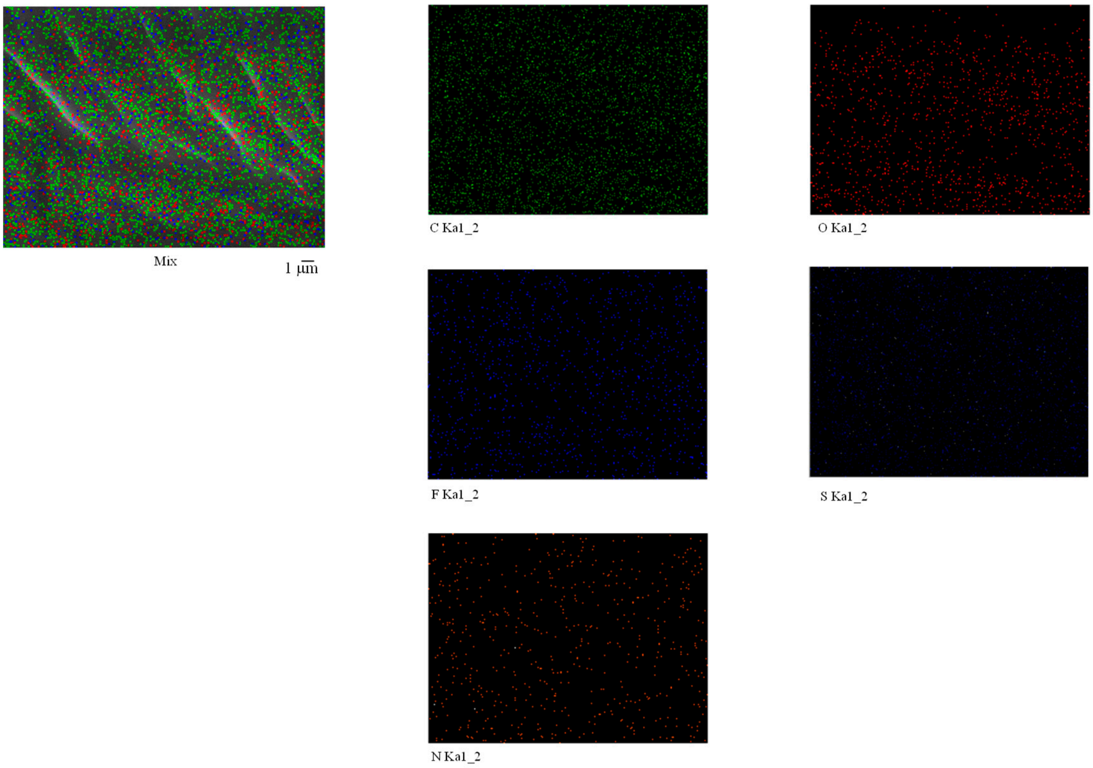

Figure S2. Scanning electron microscopy (SEM) mapping results for the [EPrI][TFSI]/PCL = 20/80 blend.

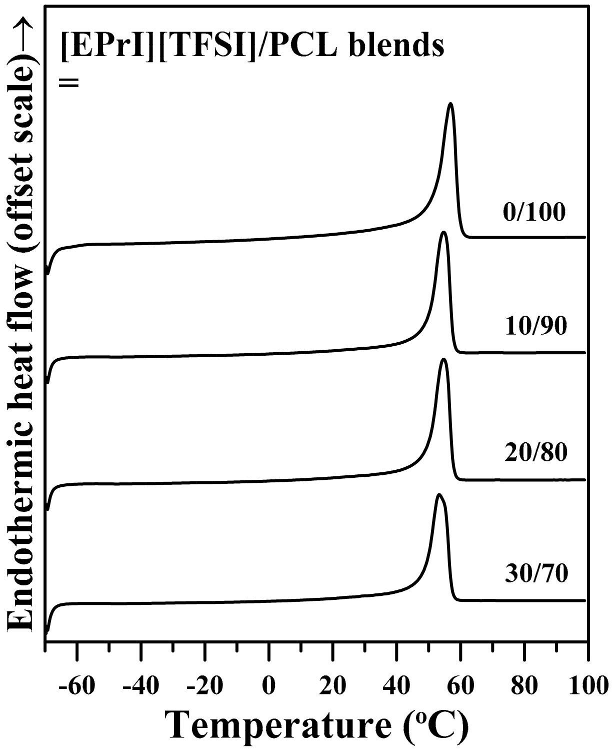

Figure S3. Differential scanning calorimetry (DSC) heating scans for the [EPrI][TFSI]/PCL blends after the melting/quenching treatment. Blends showing various compositions (wt%) were detected.

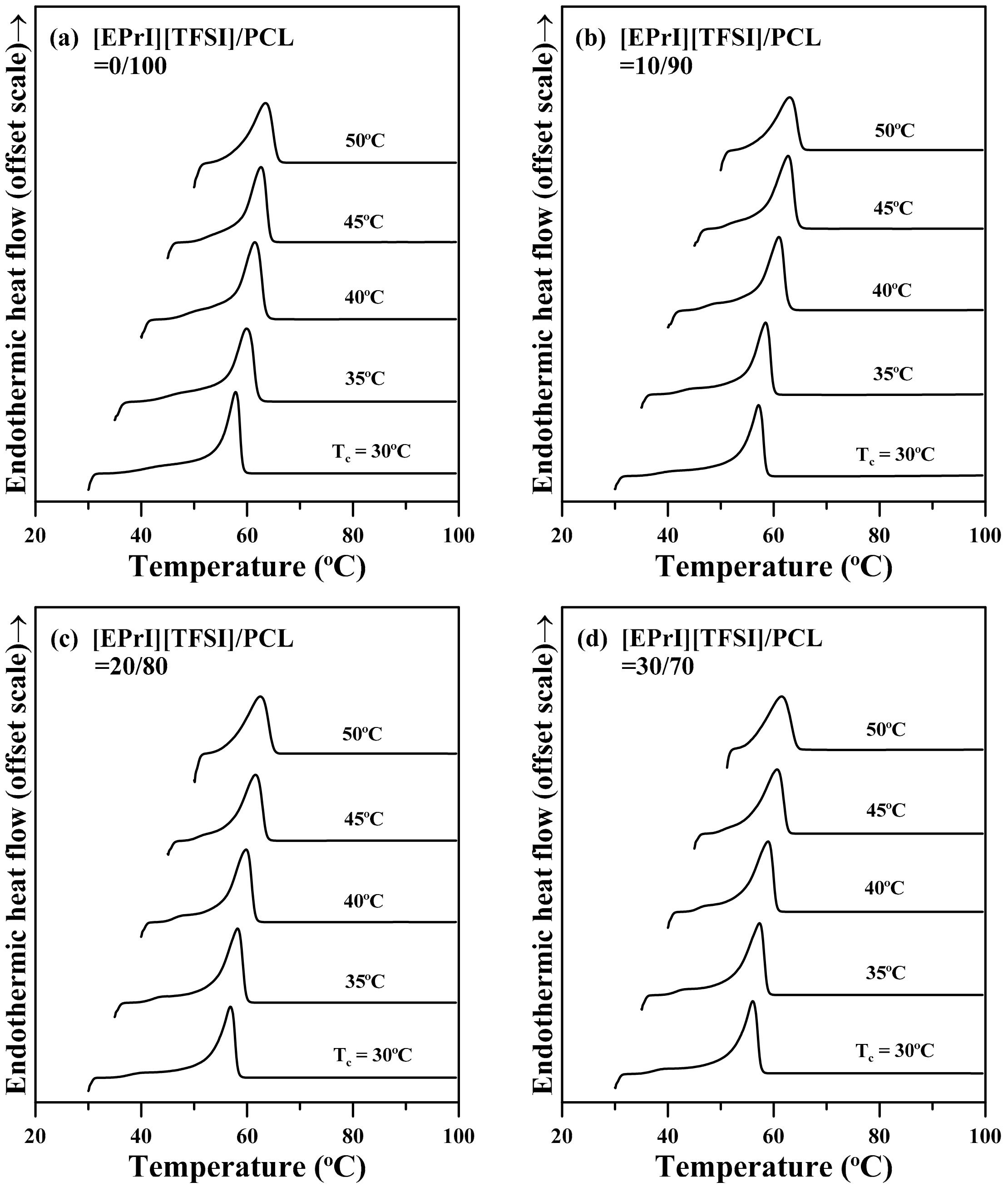

Figure S4. DSC heating traces at different T_c of the [EPrI][TFSI]/PCL blends with compositions (a) 0/100, (b) 10/90, (c) 20/80, and (d) 30/70.

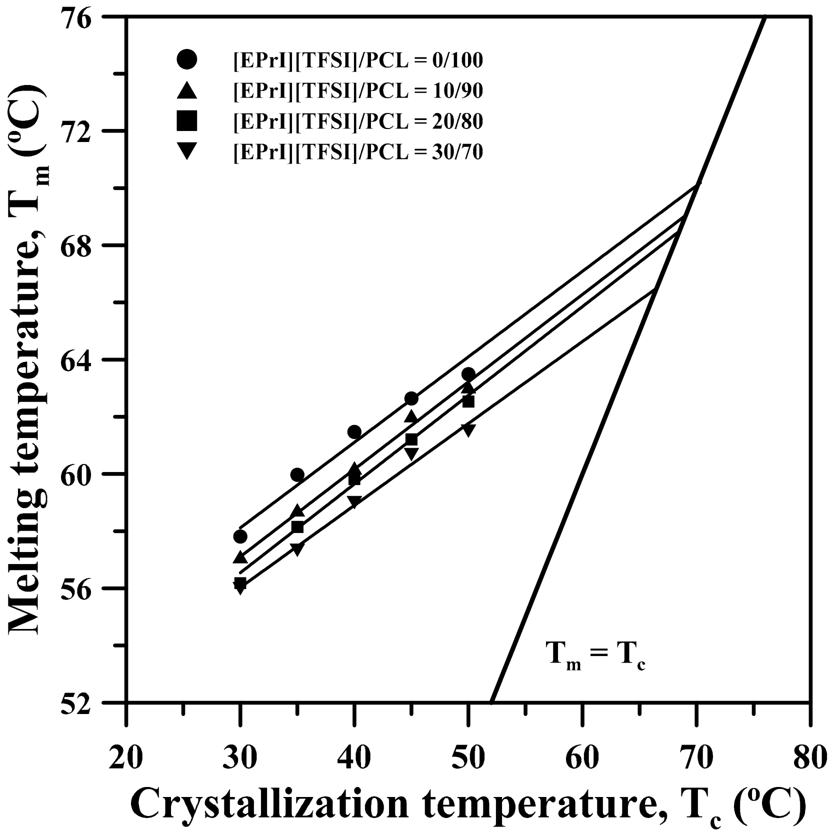

Figure S5. Hoffman–Weeks plots of the [EPrI][TFSI]/PCL blends.

Table S1. Equilibrium melting points of the [EPrI][TFSI]/PCL blends with different compositions.

[EPrI][TFSI]/PCL (wt %)	T_m^0 (°C)
0/100	70.34
10/90	69.08
20/80	68.31
30/70	66.49

References of supplementary materials:

1. Avrami, M. Kinetics of phase change II Transformation-time relations for random distribution of nuclei. J. Chem. Phys. 1940, 8, 212-224.
2. Avrami, M. Kinetics of phase change III Granulation, phase change, and microstructure. J. Chem. Phys. 1941, 9, 177-184.
3. Jeziorny, A. Parameters characterizing the kinetics of the non-isothermal crystallization of poly(ethylene terephthalate) determined by d.s.c.. Polymer 1978, 19, 1142-1144.
4. Ozawa, T. Kinetics of non-isothermal crystallization. Polymer 1971, 12, 150-158.
5. Liu, T.X.; Mo, Z.S.; Wang, S.G.; Zhang, H.F. Nonisothermal melt and cold crystallization kinetics of poly(aryl ether ether ketone ketone). Polym. Eng. Sci. 1997, 37, 568-575.
6. Friedman, H. Kinetics of Thermal degradation of char-forming plastics from thermogravimetry. application to a phenolic plastic. J. Polym. Sci. Part C, 1964, 6, 183-195.
7. Vassiliou, A.A.; Papageorgiou, G.Z.; Achilias, D.S.; Bikiaris, D.N. Non-isothermal crystallisation kinetics of in situ prepared poly(ε-caprolactone)/surface-treated SiO_2 nanocomposites. Macromol. Chem. Phys. 2007, 208, 364-376.
